# Radiopharmaceutical and Eu^3+^ doped gadolinium oxide nanoparticles mediated triple-excited fluorescence imaging and image-guided surgery

**DOI:** 10.1186/s12951-021-00920-6

**Published:** 2021-07-16

**Authors:** Xiaojing Shi, Caiguang Cao, Zeyu Zhang, Jie Tian, Zhenhua Hu

**Affiliations:** 1 CAS Key Laboratory of Molecular Imaging, Beijing Key Laboratory of Molecular Imaging, The State Key Laboratory of Management and Control for Complex Systems, Institute of Automation, Chinese Academy of Sciences, Beijing, China; 2 School of Artificial Intelligence, University of Chinese Academy of Sciences, Beijing, China; 3 Beijing Advanced Innovation Center for Big Data-Based Precision Medicine, School of Medicine, Beihang University, Beijing, China

**Keywords:** Radiopharmaceuticals, Gd_2_O_3_:Eu, Cerenkov luminescence imaging, Optical imaging, Image-guided surgery

## Abstract

Cerenkov luminescence imaging (CLI) is a novel optical imaging technique that has been applied in clinic using various radionuclides and radiopharmaceuticals. However, clinical application of CLI has been limited by weak optical signal and restricted tissue penetration depth. Various fluorescent probes have been combined with radiopharmaceuticals for improved imaging performances. However, as most of these probes only interact with Cerenkov luminescence (CL), the low photon fluence of CL greatly restricted it’s interaction with fluorescent probes for in vivo imaging. Therefore, it is important to develop probes that can effectively convert energy beyond CL such as β and γ to the low energy optical signals. In this study, a Eu^3+^ doped gadolinium oxide (Gd_2_O_3_:Eu) was synthesized and combined with radiopharmaceuticals to achieve a red-shifted optical spectrum with less tissue scattering and enhanced optical signal intensity in this study. The interaction between Gd_2_O_3_:Eu and radiopharmaceutical were investigated using ^18^F-fluorodeoxyglucose (^18^F-FDG). The ex vivo optical signal intensity of the mixture of Gd_2_O_3_:Eu and ^18^F-FDG reached 369 times as high as that of CLI using ^18^F-FDG alone. To achieve improved biocompatibility, the Gd_2_O_3_:Eu nanoparticles were then modified with polyvinyl alcohol (PVA), and the resulted nanoprobe PVA modified Gd_2_O_3_:Eu (Gd_2_O_3_:Eu@PVA) was applied in intraoperative tumor imaging. Compared with ^18^F-FDG alone, intraoperative administration of Gd_2_O_3_:Eu@PVA and ^18^F-FDG combination achieved a much higher tumor-to-normal tissue ratio (TNR, 10.24 ± 2.24 *vs.* 1.87 ± 0.73, *P* = 0.0030). The use of Gd_2_O_3_:Eu@PVA and ^18^F-FDG also assisted intraoperative detection of tumors that were omitted by preoperative positron emission tomography (PET) imaging. Further experiment of image-guided surgery demonstrated feasibility of image-guided tumor resection using Gd_2_O_3_:Eu@PVA and ^18^F-FDG. In summary, Gd_2_O_3_:Eu can achieve significantly optimized imaging property when combined with ^18^F-FDG in intraoperative tumor imaging and image-guided tumor resection surgery. It is expected that the development of the Gd_2_O_3_:Eu nanoparticle will promote investigation and application of novel nanoparticles that can interact with radiopharmaceuticals for improved imaging properties. This work highlighted the impact of the nanoprobe that can be excited by radiopharmaceuticals emitting CL, β, and γ radiation for precisely imaging of tumor and intraoperatively guide tumor resection.

**Supplementary Information:**

The online version contains supplementary material available at 10.1186/s12951-021-00920-6.

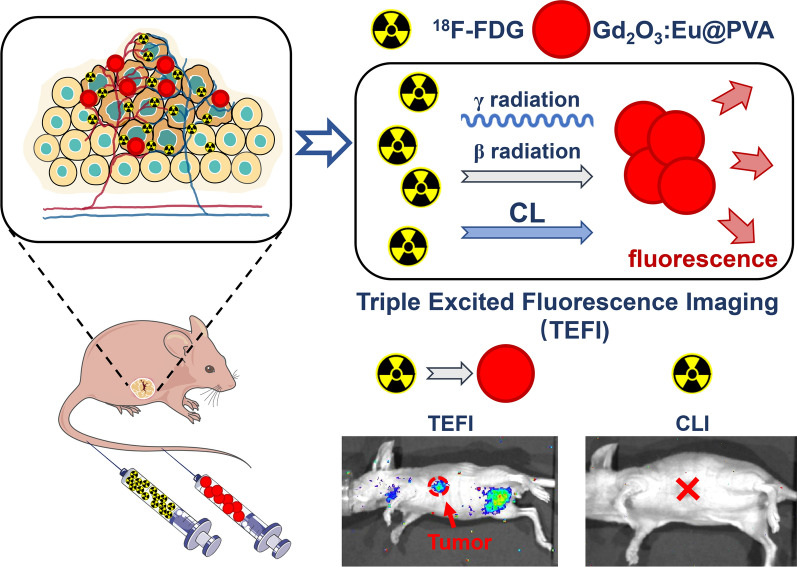

## Introduction

Cerenkov luminescence imaging (CLI) is an important optical imaging technique based on Cerenkov radiation generated along with the decay process of various radionuclides [1–4]. Numerous Food and Drug Administration (FDA)-approved radiopharmaceuticals that were originally used for positron emission computed tomography (PET) can generate Cerenkov luminescence (CL), providing CLI with high potential for clinical translation. Therefore, since the first biomedical imaging of small animals in 2009, CLI-based tumor imaging and image-guided tumor resection surgery have been broadly investigated in pre-clinical applications and also explored in clinic [5–14]. The combination of CLI and PET enables surgeon to achieve the distribution of the same imaging agent before and during surgery, which may provide surgeon with more information on the tumor location and improve the accuracy of the tumor resection surgery [15, 16]. However, as optical signal intensity of CL is extremely weak, a long exposure time is often used in image acquisition. Besides, the ultraviolet-blue spectrum of the CL restricted penetration. These limit the application of CLI in intraoperative real-time tumor imaging.

Various fluorescent probes including small-molecule agents and nanoparticle (NP) probes have been combined with radiopharmaceuticals for improved imaging performances. Research has been focused on quantum dots (QDs) that can interact with CL, achieving emitted light in the near-infrared range with deeper tissue-penetration. This imaging technique was named as radiation excited luminescence imaging [17] or the secondary Cerenkov emission fluorescence imaging (SCIFI) [18]. Another research has demonstrated that a clinically available imaging agent, fluorescein sodium (FS) which has been commonly used for retinal blood vessel imaging, can also be excited by Cerenkov photons for surgical navigation [19]. However, low photon fluence of CL greatly restricted it’s interaction with fluorescent probes for in vivo imaging [20]. Therefore, novel imaging probes that can interact with radiopharmaceuticals through additional mechanisms have been desired. It was reported that the β particles and γ radiation generated along with the decay process of the radiopharmaceutical can also interact with some NPs [17, 21, 22]. These interactions can result in the ionization of the NP, with the fluorescence of higher signal intensity and longer wavelength emitted as the NP relaxes to the baseline state [23–27]. Therefore, the europium oxide (Eu_2_O_3_) NP that can be excited by CL and interact with γ radiation as well has been developed to achieve an enhanced optical intensity and red-shifted optical spectrum of radiopharmaceuticals, which improves the tumor-to-normal tissue ratio (TNR) and shortens the exposure time [26–28]. Recently, ZnGa_2_O_4_:Cr^3+^ NPs with persistent luminescence were reported to be activated by radiopharmaceuticals. The persistent luminescence of the NPs enabled long-lasting tumor detecting with high sensitivity and contrast [29, 30].

In this work, novel Eu^3+^ doped gadolinium oxide (Gd_2_O_3_:Eu) NPs were synthesized to be combined with radiopharmaceuticals for improved imaging performance. The commonly used clinical radiopharmaceutical 2-deoxy-2-^18^F-fluoroglucose (^18^F-FDG) was used to provide CL, β particles, and γ radiation. By mixing ^18^F-FDG and Gd_2_O_3_:Eu NPs, an enhanced red-shifted emission light was achieved. It was found that Gd_2_O_3_:Eu NPs interact with CL, β, and γ radiation, which turned the energy of radiopharmaceuticals into fluorescence with high efficiency. Therefore, the imaging method was named as triple-excited fluorescence imaging (TEFI). Moreover, Gd_2_O_3_:Eu NPs were modified by polyvinyl alcohol (PVA) for improvement of biocompatibility. In the end, performance of PVA modified Gd_2_O_3_:Eu (Gd_2_O_3_:Eu@PVA) on tumor imaging and image-guided surgery were evaluated using subcutaneous breast tumor-bearing mouse models. It was demonstrated that Gd_2_O_3_:Eu@PVA and ^18^F-FDG combination improved intraoperative tumor detection with a high imaging contrast.

## Materials and methods

### Synthesis of Gd_2_O_3_:Eu NPs

The hydrothermal method was used in the synthesis of Gd_2_O_3_:Eu NPs referring to the previous report [31]. 1.52 mmol Gd(NO_3_)_3_·9H_2_O, 0.08 mmol Eu(NO_3_)_3_·6H_2_O, and 0.2168 g urea were mixed with 8 mL of deionized water (DI water) and different amount of glycerol. The mixture was stirred until the solution turned clear and was then transferred into a stainless-steel reactor. The reaction lasted 500 min under 160 ℃. After the reaction, the solution was cooled and centrifugated. The product was washed with DI water three times. The product was then dried using a lyophilizer at –60 ℃ under vacuum. The dried precipitate was calcinated at 1000 ℃ for another 4 h. The dose of glycerol was regulated to achieve Gd_2_O_3_:Eu NPs of different sizes. Glycerol (3, 1, or 0.5 mL) was added to obtain Gd_2_O_3_:Eu with a diameter of 50, 100, and 200 nm (named as Gd_2_O_3_:Eu-50, Gd_2_O_3_:Eu-100, and Gd_2_O_3_:Eu-200), respectively.

### PVA modification of NPs

To modify the NPs with PVA, Gd_2_O_3_:Eu-50, Gd_2_O_3_:Eu-100, and Gd_2_O_3_:Eu-200 were added into the DI water solution of PVA separately. The mixture of the Gd_2_O_3_:Eu NPs and PVA were stirred using quartz beads to ensure that the Gd_2_O_3_:Eu NPs were uniformly coated with PVA and formed Gd_2_O_3_:Eu-50, Gd_2_O_3_:Eu-100, and Gd_2_O_3_:Eu-200 modified with PVA (Gd_2_O_3_:Eu-50@PVA, Gd_2_O_3_:Eu-100@PVA, and Gd_2_O_3_:Eu-200@PVA). The mixture was then dried using a lyophilizer at -60 ℃ under vacuum.

### Characterization of Gd_2_O_3_:Eu and Gd_2_O_3_:Eu@PVA NPs

The size and morphology of Gd_2_O_3_:Eu NPs were tested by Transmission Electron Microscope (TEM, JEOL Ltd, Tokyo, Japan). The diameters of the NPs were measured with Image J according to the TEM images (National Institutes of Health, Maryland, USA). Fluorescent properties including emission and excitation spectra were measured using EnSpire Multimode Plate Readers (PerkinElmer, Inc., Massachusetts, USA). The crystal characteristics of the nanoparticles were tested with an Ultima IV X-ray diffractometer (XRD, Rigaku, Tokyo, Japan). X-ray photoelectron spectroscopy (XPS) measurements were carried out with Thermo Scientific K-Alpha + (Thermo Fisher Scientific, Massachusetts, USA).

### Fluorescence imaging

The fluorescence images were acquired with the IVIS Spectrum imaging system (PerkinElmer, Inc., Massachusetts, USA). In the experiments on the influencing factors of optical signal intensity of Gd_2_O_3_:Eu NPs, an exposure time of 60 s was adopted. While in the in vitro experiments to verify the interaction type, an exposure time of 300 s was adopted. To evaluate the spectrum of the emitted light, a series of bandpass filters with a discrete center wavelength from 500 to 840 nm integrated into the IVIS system was adopted with an exposure time of 20 s. For the experiments on tissue penetration, an open filter was applied, with the exposure time set to be 5 s. In the animal experiments of phantom study, imaging, and image-guided surgery, an open filter and an exposure time of 300 s were used.

### Investigation of the factors affecting radical interaction

To select the most suitable NP for biomedical imaging, 5 mg Gd_2_O_3_:Eu-50, Gd_2_O_3_:Eu-100, Gd_2_O_3_:Eu-200, Gd_2_O_3_:Eu-50@PVA, Gd_2_O_3_:Eu-100@PVA, or Gd_2_O_3_:Eu-200@PVA were set in 6 different Eppendorf (EP) tubes and mixed with ^18^F-FDG (430 μCi, 100 μl), respectively. Images of the 6 EP tubes were acquired to evaluate the signal intensity. Gd_2_O_3_:Eu and Gd_2_O_3_:Eu@PVA with a diameter of 100 nm were selected for the following ex vivo and in vivo experiments based on the aforementioned experimental results.

To investigate the impact of distance between the excitation source (^18^F-FDG) and the NP (Gd_2_O_3_: Eu) on the optical intensity produced, a single well of a transparent 96-well plate loaded with ^18^F-FDG (730 μCi, 100 μl), and an EP tube containing Gd_2_O_3_:Eu-100 (20 mg) was placed with the distance between the bottom of the tube and the well set to be 10, 20, 30, 40, and 50 mm. The signal intensity of Gd_2_O_3_:Eu-100 NP on each image was measured, and its correlation with distance was then determined.

To evaluate impact of radioactivity, a series of EP tubes containing 100 μl of ^18^F-FDG with different radioactivity (1128, 552, 285, 145, 73, 36, 18, 9, 4, and 2 μCi) were applied as excitation source successively. Gd_2_O_3_:Eu-100 (20 mg) was placed in another EP tube. The distance between them was set to be 10 mm. The signal intensity of the Gd_2_O_3_:Eu-100 NP was measured and correlated to the radioactivity of the ^18^F-FDG in each image.

To assess the impact of mass, ^18^F-FDG (730 μCi, 100 μl) was set in a well of a transparent 96-well plate. Gd_2_O_3_:Eu-100 with different mass (20, 10, 5, 2.5, 1 mg) was placed in EP tubes, respectively. The distance between the well and the tubes was 10 mm. the signal intensity of each EP tube containing Gd_2_O_3_:Eu-100 with different mass was measured and correlated to the mass.

Eu_2_O_3_ of the same mass with Gd_2_O_3_: Eu was used as a comparison in each experiment as stated above.

### Investigation of interaction mechanisms between Gd_2_O_3_:Eu and ^18^F-FDG

To reveal the mechanisms that result in the emission of light, Gd_2_O_3_:Eu-100 powder (20 mg) and ^18^F-FDG (2.2 mCi, 100 μl) were placed at the bottom of two EP tubes. Images were first acquired when the bottom of the two EP tubes was placed next to each other, with no blocking between them. Therefore, the CL, β particle and γ radiation generated from ^18^F-FDG can all interact with Gd_2_O_3_:Eu-100. An aluminum plate that blocked CL and β particles and a lead plate that blocked CL, β particles, and γ radiation were then placed between the two tubes in order. Black tapes were then used to cover the EP tube containing ^18^F-FDG to block CL only. Images were acquired in each step. Besides, to further investigate the contribution of CL to the emission, the two EP tubes containing ^18^F-FDG and Gd_2_O_3_:Eu-100 were placed with the bottom 20 mm apart. Two mirrors were placed on both sides of the tubes to reflect CL. Optical images were acquired to reveal the three types of interaction with CL, β particle, and γ radiation. With a lead plate set between the two tubes, β particles and γ radiation were blocked, where only CL can interact with the Gd_2_O_3_:Eu-100. Optical images were then acquired for evaluation of light emission caused by CL. The same operation was repeated with Gd_2_O_3_:Eu-100 replaced by Eu_2_O_3_ powder (20 mg) to investigate the contribution of CL to the emission when the Eu_2_O_3_ NP was used.

### Assessment of emission spectrum and tissue penetration ability of the light

Gd_2_O_3_:Eu, Gd_2_O_3_:Eu@PVA of different sizes (50, 100, and 200 nm) and Eu_2_O_3_ (powder, each 5 mg) were mixed with ^18^F-FDG (430 μCi, 100 μl) separately in a well of the 96-well plate. Saline solution (100 μl) and ^18^F-FDG (430 μCi, 100 μl) were used as the control. The penetration ability of the light emitted from the mixtures in biological tissue was then evaluated using a porcine intestine covering the 96-well plate.

### Cell culturing and animal model establishment

All the experimental procedures involving animals were approved by the Institutional Animal Care and Use Committee of the Fifth Affiliated Hospital, Sun Yat-sen University (2020071401). 4T1 mouse mammary tumor cells were cultured with RPMI 1640 medium (Gibco, Life Technologies, Carlsbad, CA) supplemented with 10% fetal bovine serum (FBS, Gibco, Life Technologies, Carlsbad, CA), 100 U/mL penicillin and 100 μg/ml streptomycin (Life Technologies, Carlsbad, CA) in a humidified incubator at 37 °C with 5% CO_2_.

Female balb/c nude mice of 4 weeks (Beijing Vital River Laboratory Animal Technology Co. Ltd, Beijing, China) were used in this study. The subcutaneous breast cancer mice models were established by injecting 5 × 10^6^ 4T1 mouse mammary tumor cells subcutaneously in mice. Seven days after injection, the mice were used for imaging and image-guided surgery experiment. Animal surgery and imaging were performed under isoflurane gas anesthesia (3% isoflurane and air mixture), and all the possible actions were employed to minimize the suffering of the mice.

### In vivo optical imaging using capillary phantoms

Two capillaries were subcutaneously embedded into the back of the athymic nude mouse. One capillary was filled with ^18^F-FDG (84 μCi, 20 μl), while the other was filled with the mixture of Gd_2_O_3_:Eu@PVA (2 mg) and ^18^F-FDG (84 μCi, 20 μl). PET and optical imaging were performed for comparison.

### In vivo evaluation of the Gd_2_O_3_:Eu-100@PVA and ^18^F-FDG mixture

To study the performance of Gd_2_O_3_:Eu-100@PVA in improving the optical signal intensity of ^18^F-FDG in vivo, breast tumor-bearing mice (n = 8) were randomly assigned to the TEFI and CLI group (n = 4 for each group). For the TEFI group, mice were injected intravenously with ^18^F-FDG (250 μCi, 200 μl) and Gd_2_O_3_:Eu-100@PVA (1 mg/ml, 100 μl), while the mice in the CLI group were injected with only ^18^F-FDG (1 mg/ml, 100 μl). Imaging was performed 1.5 h after injection using the IVIS system.

### PET imaging of small animals

Static 5-min PET images of animals injected with ^18^F-FDG were acquired using a preclinical PET/CT scanner (Genisys PET, SofieBiosciences, Inc., USA). The data acquisition mode of ^18^F was integrated into the device.

### Biodistribution of Gd_2_O_3_:Eu@PVA

The tumor-bearing mice in the TEFI group were euthanized immediately after NPs based optical imaging. The tumor, heart, liver, spleen, lung, kidney, intestine, brain, and muscle were harvested, and blood was also collected for ex vivo optical imaging.

## Results

### The characteristic of the synthesized Gd_2_O_3_:Eu NPs

**Fig. 1:**
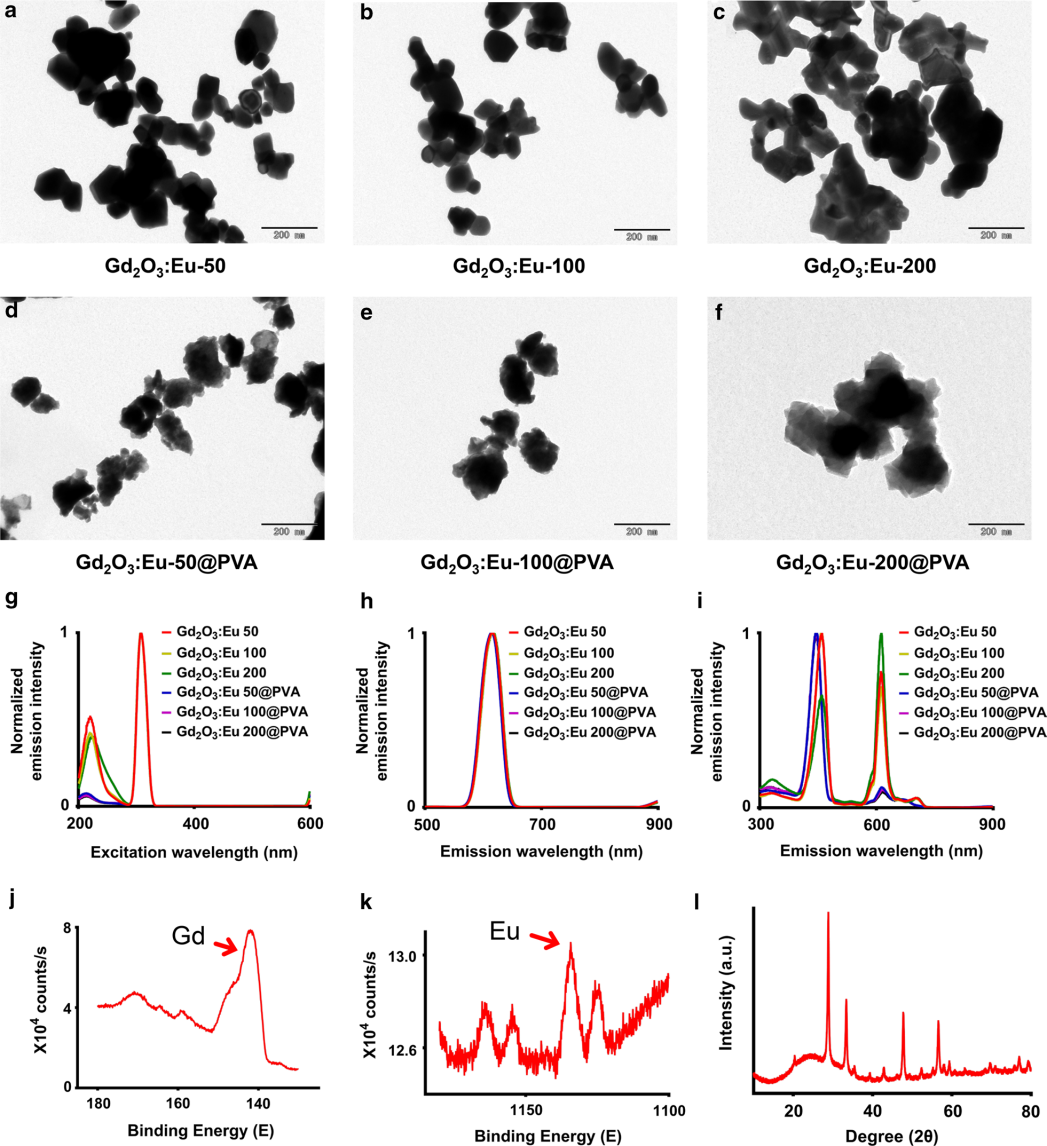
Nanoparticle morphology and spectrum. **a**–**f** the TEM images of the NPs with different sizes and surface modification. Scale bar, 200 nm. **g** the excitation peaks lays on 214 and 308 nm, keeping the emission fixed at 620 nm. **h** an emission peak of 620 nm was displayed under 214 nm excitation. **i** emission peaks of 460 nm, 620 nm, and 700 nm were displayed under 308 nm excitation light. **j**, **k** the XPS spectrum of Gd (**j**) and Eu (**k**) from the Gd_2_O_3_:Eu-100 NP. **l** XRD patterns of the Gd_2_O_3_:Eu-100 NP

The size of Gd_2_O_3_:Eu-50, Gd_2_O_3_:Eu-100, and Gd_2_O_3_:Eu-200 NPs was in ranges of 50–75, 90–110, and 170–230 nm, respectively (Fig. 1a–c), as measured on the TEM images. The surface modification with PVA did not significantly change the size and morphology of Gd_2_O_3_:Eu NPs, with the diameters laid in ranges of 65–88, 95–116, and 195–244 nm (Fig. 1d–f).

The excitation spectrum showed that the maximum excitation wavelength of the NPs, including Gd_2_O_3_:Eu and Gd_2_O_3_:Eu@PVA with different diameters (50,100, 200 nm) was 308 nm, with a smaller peak laying on around 214 nm (Fig. 1g). When excited by 308 nm excitation light, the emission peak was 620 nm for all the NPs (Fig. 1h). When excited by 214 nm excitation light, the emission spectrum had three peaks lying on 460, 620, and 700 nm (Fig. 1i).

The peaks of the XPS spectrum at the 141.98 and 1134.48 eV binding energy proved the presence of the Gd and Eu (Fig. 1j, k). The XRD spectrum of the Gd_2_O_3_:Eu-100 NP was matched with the standard JCPDS 12–0797 card. The XRD peaks were related only to the Gd_2_O_3_ nanoparticles (Fig. 1l).

### The influential factors of optical signal intensity

**Fig. 2:**
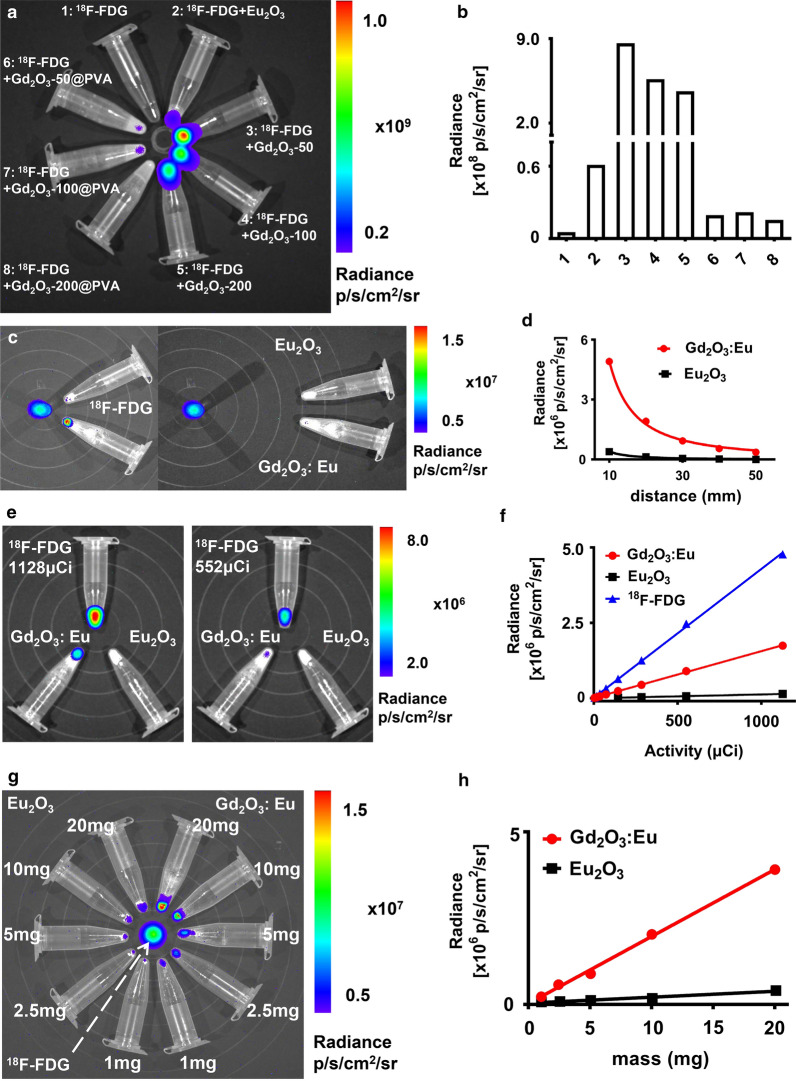
The factors impacting the optical signal intensity of interaction**. a,b,** optical images (**a**) and quantitative analysis (**b**) displayed that the size and surface modification of the NPs affected the optical signal intensity. **c**, **d** the optical signal intensity is in inverse proportion to the interaction distance between Gd_2_O_3_:Eu-100/ Eu_2_O_3_ and ^18^F-FDG. **e**,** f** the optical signal intensity is in direct proportion to the radioactivity of ^18^F-FDG. **g**,** h** the optical signal intensity is in direct proportion to the mass of the Gd_2_O_3_:Eu/ Eu_2_O_3_

The optical signal intensity of the NPs was affected by the particle size and surface modification (Fig. 2a, b). For Gd_2_O_3_:Eu without surface modification of PVA, the optical signal intensity decreased as the particle size increased, with the Gd_2_O_3_:Eu-50 generating light with the highest optical signal intensity and Gd_2_O_3_:Eu-200 generating light with the lowest optical signal intensity. For Gd_2_O_3_:Eu with surface modification by PVA, Gd_2_O_3_:Eu-100@PVA generated light with the highest optical signal intensity while Gd_2_O_3_: Eu-200@PVA generated light with the lowest optical signal intensity. As the mass of the particles used were kept the same, the amount of the Gd_2_O_3_:Eu may be replaced by PVA. Therefore the optical signal was influenced by PVA modification though it may improve biocompatibility. With the results obtained above, the 100 nm NPs were further evaluated in the following experiments, with Gd_2_O_3_:Eu-100@PVA used in the in vivo experiments and Gd_2_O_3_:Eu-100 tested in the ex vivo experiments.

The influencing factors of the optical signal intensity also included excitation distance, the amount of the radioactivity of ^18^F-FDG, and the mass of NPs (Gd_2_O_3_:Eu-100 and Eu_2_O_3_). The optical signal intensity decreased exponentially as the excitation distance increased for both Gd_2_O_3_:-Eu-100 and Eu_2_O_3_, with R^2^ of 0.9972 for Gd_2_O_3_:Eu-100 and 0.9931 for Eu_2_O_3_ (Fig. 2c, d, Additional file 1: Fig. S1a). The optical signal intensity of the NP increased linearly with the increasing of the radioactivity of ^18^F-FDG, with R^2^ of 0.9994 for Gd_2_O_3_:-Eu and 0.9998 for Eu_2_O_3_ (Fig. 2e, f, Additional file 1: Fig. S1b). As for the mass of NPs, the optical signal intensity of the NPs also increased linearly with the mass of NPs, with R^2^ of 0.9975 for Gd_2_O_3_:Eu and 0.9737 for Eu_2_O_3_ (Fig. 2g, h). The optical signal of Gd_2_O_3_:Eu was much higher than that of Eu_2_O_3_ in each of the studies.

### Investigation of the optical signal caused by CL, β particles, and γ radiation of ^18^F-FDG

**Fig. 3:**
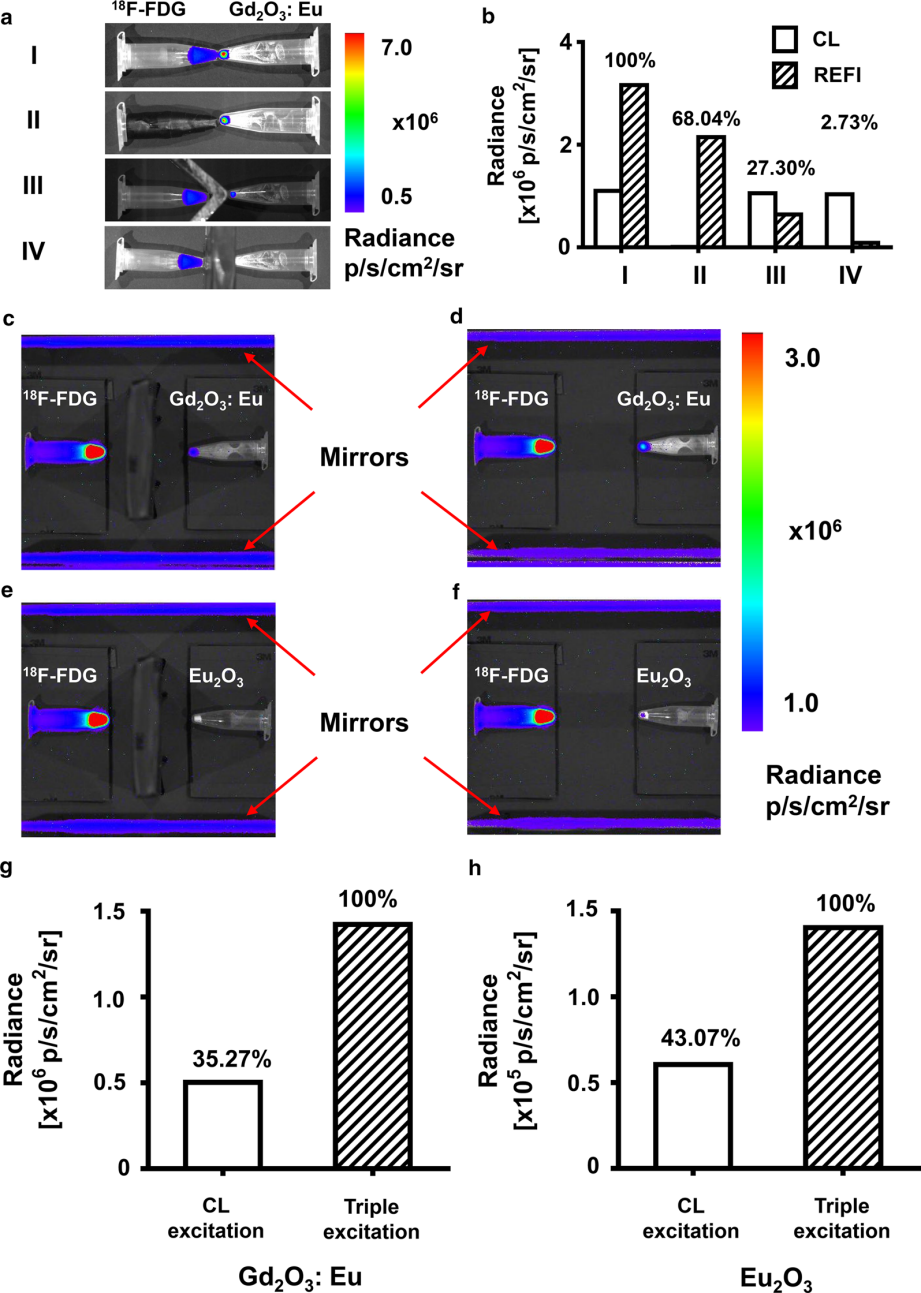
The investigation on the excitation mechanism**. a** the overlayed images of the optical signal generated by Gd_2_O_3_:Eu-100. **b** the optical signal intensity of each tube. **c**–**f** the images of the optical signal generated by Gd_2_O_3_:Eu-100 through interaction with CL, with β particles and γ radiation blocked by the lead plate and CL reflected by the mirrors (**c**), by Gd_2_O_3_:Eu-100 through interaction with CL, β particles, and γ radiation (**d**), by Eu_2_O_3_ through interaction with CL (**e**) and by Eu_2_O_3_ through interaction with CL, β particles, and γ radiation (**f**). **g**, **h** the optical signal intensity of each condition for Gd_2_O_3_:Eu-100 (**g**) and Eu_2_O_3_ (**h**)

It was observed that emission light was generated by Gd_2_O_3_:Eu 100 through interactions with CL, β particle, and γ radiation generated by ^18^F-FDG (Fig. 3a). The overall optical signal intensity caused by all three types of interactions was set to be 100% (Fig. 3a row I and Fig. 3b I). With only CL blocked by black tape, the optical signal caused by interaction with β particles and γ radiation was observed, which accounted for 68.04% of the overall optical signal intensity (Fig. 3a row II and Fig. 3b II). With CL and β particles both blocked by an aluminum plate, the optical signal caused by interaction with γ radiation was acquired, which only accounted for 27.30% of the overall optical signal intensity (Fig. 3a row III and Fig. 3b III). In the further experiment where CL, β, and γ radiation were all blocked by a lead plate, the optical signal was barely acquired (Fig. 3a row IV and Fig. 3b IV). It was calculated that 31.97% of the optical signal was caused by CL, 40.74% of the optical signal was caused by β particles, and 27.30% was caused by γ radiation. Moreover, with β particles and γ radiation blocked by a lead plate, but CL reflected by two mirrors, the optical signal caused by interaction with CL was evaluated independently (Fig. 3c–f). For Gd_2_O_3_:Eu-100, 35.27% of the optical signal was caused by CL, which was in accordance with the result of the experiments above (Fig. 3c, d). For Eu_2_O_3_, the previously reported radiopharmaceutical excitable NP, 43.07% of the optical signal was caused by CL according to measurement (Fig. 3e, f).

### Characterization and tissue penetration of the red-shifted emission light

**Fig. 4:**
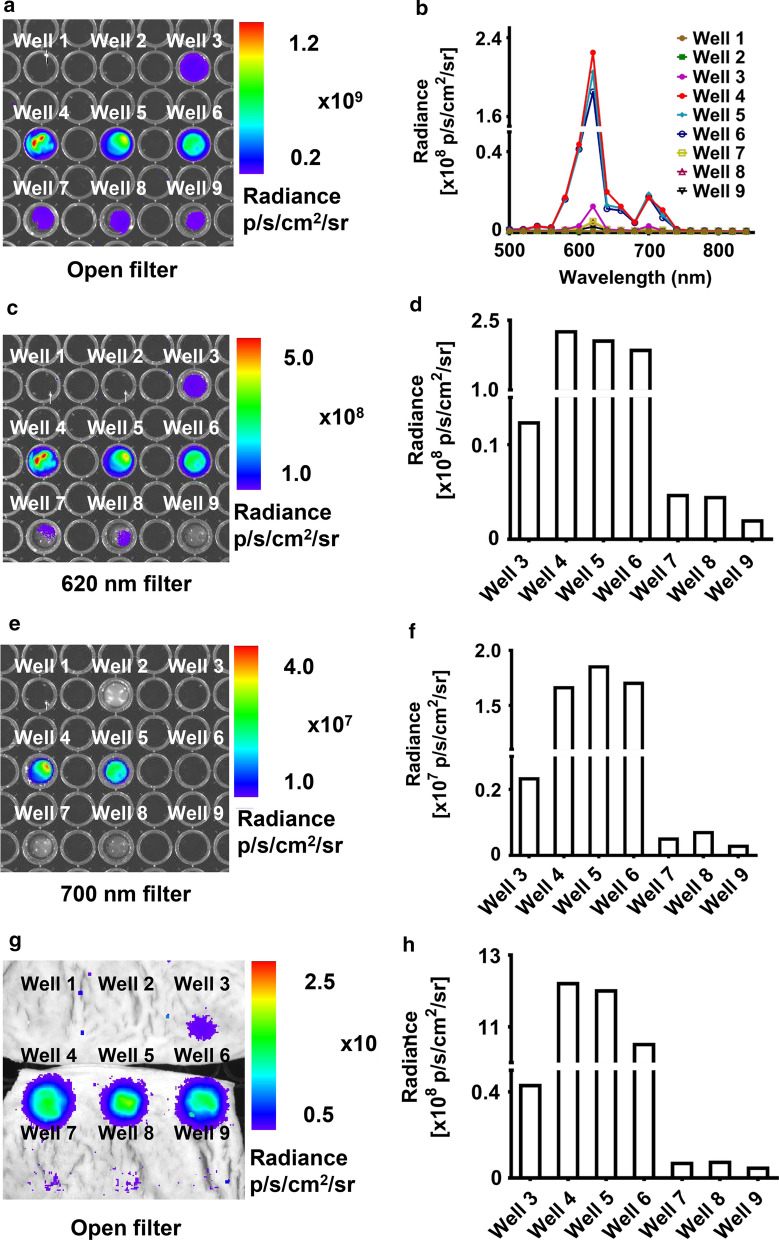
The profile of optical imaging of the Gd_2_O_3_:Eu and ^18^F-FDG mixture**.** The contents in the Well 1–9 in this figure are listed as follows: Well 1: saline solution. Well 2: ^18^F-FDG. Well 3: Eu_2_O_3_ + ^18^F-FDG. Well 4: Gd_2_O_3_:Eu-50 + ^18^F-FDG. Well 5: Gd_2_O_3_:Eu-100 + ^18^F-FDG. Well 6: Gd_2_O_3_:Eu-200 + ^18^F-FDG. Well 7: Gd_2_O_3_:Eu-50@PVA + ^18^F-FDG. Well 8: Gd_2_O_3_:Eu-100@PVA + ^18^F-FDG. Well 9: Gd_2_O_3_:Eu-200@PVA + ^18^F-FDG. **a** optical imaging with an open filter. **b** the emission spectrum of contents in each well. **c**,** d** optical image (**c**), and quantification of the optical signal intensity (**d**) with 620 nm filter. **e**, **f** optical image (**e**), and quantification of the optical signal intensity (**f**) with 700 nm filter. **g**, **h** optical image (**g**), and quantification of the optical signal intensity (**h**) with swine intestine covered on the top

The 5 mg NPs (Eu_2_O_3,_ Gd_2_O_3_:Eu, and Gd_2_O_3_:Eu@PVA) extensively enhanced the optical signal intensity and tissue penetration capacity of ^18^F-FDG (Fig. 4). The optical image acquired with an open filter demonstrated that the signal intensity of the mixture of Gd_2_O_3_: Eu NPs (with diameters of 50, 100, and 200 nm) and ^18^F-FDG were all higher than that of ^18^F-FDG alone and the mixture of Eu_2_O_3_ and ^18^F-FDG. It was demonstrated that the optical signal intensity of the novel Gd_2_O_3_:Eu-50 NPs was 16.19 times higher than that of Eu_2_O_3_ when mixed with ^18^F-FDG, and reached 369 times higher than that of ^18^F-FDG alone (Fig. 4a). The emission peak of the emission light measured using bandpass filters also laid on 620 and 700 nm, which was in accordance with the spectrum measured using a spectrometer previously (Fig. 4b). Images acquired with a 620 nm filter demonstrated that the optical signal intensity of Gd_2_O_3_:Eu-50 was the highest, followed by the Gd_2_O_3_:Eu-100, and Gd_2_O_3_:Eu-200 (Fig. 4c, d). While mentioning the Gd_2_O_3_:Eu NPs modified by PVA, it was the same case that Gd_2_O_3_:Eu-50@PVA possessed the emission light with the highest intensity, followed by Gd_2_O_3_:-Eu-100@PVA and Gd_2_O_3_:Eu-200@PVA (Fig. 4c, d). However, when it came to the 700 nm filter, the Gd_2_O_3_:Eu-100 possessed the highest optical signal intensity (Fig. 4e, f). Therefore, for Gd_2_O_3_:Eu with surface modification, with the relatively weak optical signal of 620 nm wavelength, the accumulated optical signal of a broad spectrum of Gd_2_O_3_:Eu-100@PVA was the highest among Gd_2_O_3_:Eu NPs with surface modification.

With a porcine intestine covered on the top, the CL of the ^18^F-FDG was nearly blanketed and almost unmeasurable. Whereas the optical signal of the NPs and ^18^F-FDG mixtures were rather higher. The Gd_2_O_3_:Eu-50 showed the highest optical signal intensity among the three Gd_2_O_3_:Eu-NPs with different diameters. While Gd_2_O_3_:-Eu-100@PVA showed the highest optical signal intensity among the three Gd_2_O_3_:Eu NPs with surface modification (Fig. 4g, h).

### Validation using in vivo capillary phantom and living animal models

**Fig. 5:**
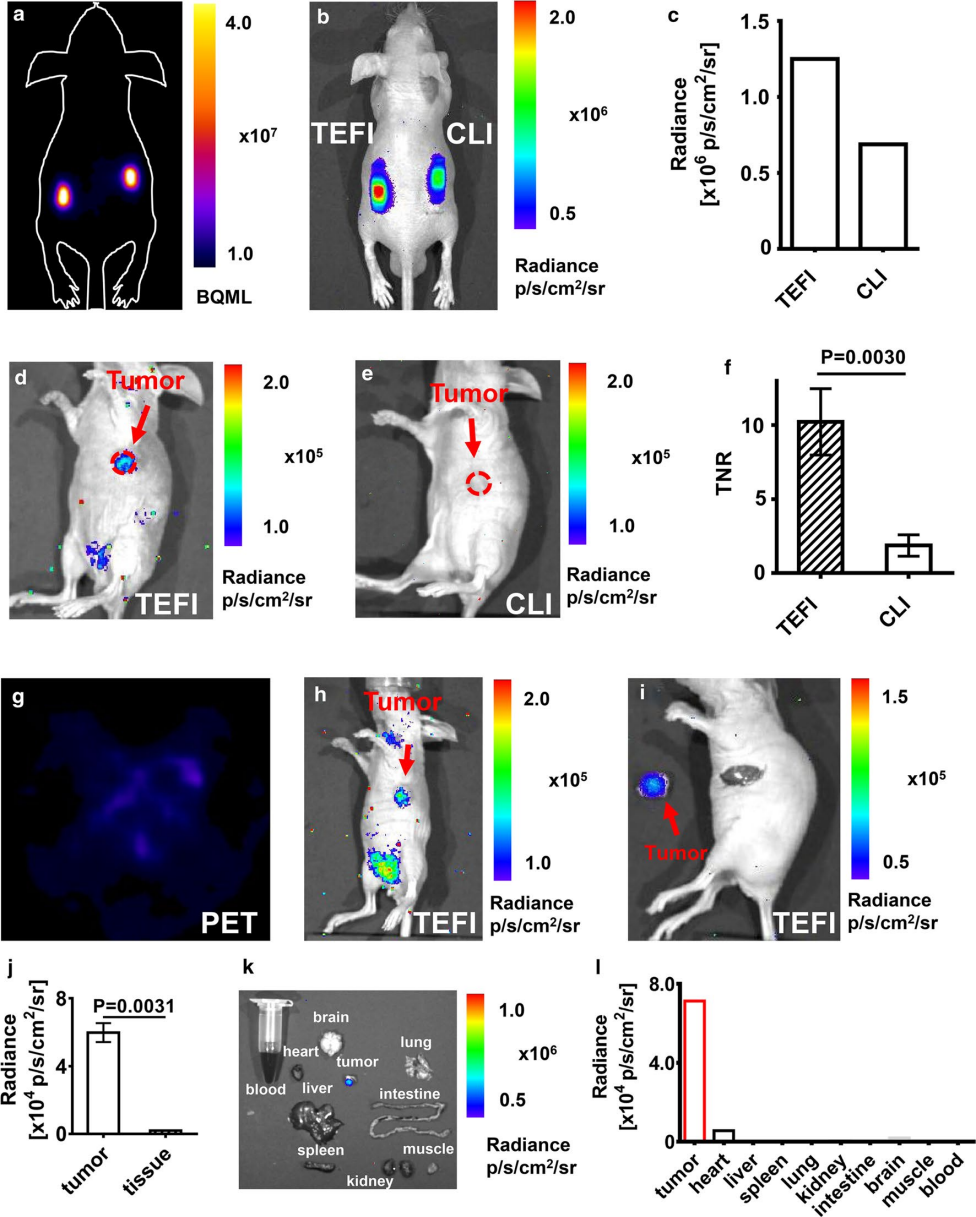
In vivo phantom and animal experiments. **a** the PET image of the animal phantom showed the same radioactivity of ^18^F-FDG in the capillaries. **b**, **c** the optical image and the quantitative analysis showed a higher signal intensity of the mixture of Gd_2_O_3_:Eu-100@PVA and ^18^F-FDG compared with ^18^F-FDG alone (left: ^18^F-FDG (84 μCi, 20 μl) + Gd_2_O_3_:Eu@PVA (2 mg), right: ^18^F-FDG (84 μCi, 20 μl)). **d** Gd_2_O_3_:Eu-100@PVA enhanced the optical signal of ^18^F-FDG, showing an obvious tumor optical signal. **e** CLI displayed no obvious signal of the tumor. **f** the TNR of the tumor was also increased by the Gd_2_O_3_:Eu-100@PVA NP. **g** the PET image of the tumor-bearing mouse injected the mixture of Gd_2_O_3_:Eu-100@PVA and ^18^F-FDG displayed no obvious tumor signal. **h** the Gd_2_O_3_: Eu 100@PVA enhanced the optical signal of the tumor. **i** the tumor was resected with the guidance of the optical image. **j** the optical signal intensity of the tumor was significantly higher than that of the surrounding normal tissue. **k**, **l** the optical image, and the quantification showed the significantly higher optical signal intensity of tumor compared with muscles and other organs obtained from the tumor-bearing mouse

With the living phantom established using capillaries that contained ^18^F-FDG or ^18^F-FDG mixed with Gd_2_O_3_:Eu-100@PVA, the feasibility of the in vivo use of Gd_2_O_3_:Eu-100@PVA NP was investigated. The PET image showed an equal signal intensity of the two implanted tubes, indicating similar radioactivity of ^18^F-FDG in the two capillaries (Fig. 5a). Nevertheless, the optical signal intensity of the ^18^F-FDG and Gd_2_O_3_:Eu-100@PVA mixture was enhanced twice of the intensity of ^18^F-FDG alone upon measurement (Fig. 5b, c).

For in vivo imaging, Gd_2_O_3_:Eu-100@PVA combined with ^18^F-FDG provided a higher tumor imaging contrast compared with CLI using ^18^F-FDG alone (Fig. 5d, e). The optical signal of the tumor was not observed using CLI (Fig. 5d). However, with Gd_2_O_3_:Eu-100@PVA injected together with ^18^F-FDG, the optical signal of the tumor was visualized with high contrast using TEFI (Fig. 5e). The tumor-to-normal tissue ratio (TNR) of the Gd_2_O_3_:Eu-100@PVA and ^18^F-FDG was significantly higher than that of CLI (10.24 ± 2.24 *vs.* 1.87 ± 0.73, *P* = 0.0030, Fig. 5f).

### Triple-excited fluorescence (TEF) image-guided tumor surgery and biodistribution

For the mice models injected with ^18^F-FDG and Gd_2_O_3_:Eu-100@PVA, the PET images acquired 1.5 h after injection showed no obvious tumor signal (Fig. 5g). However, the TEF images showed an obvious tumor signal (Fig. 5h). The tumor was then resected under the guidance of TEF images, with an ex vivo optical image of the tumor demonstrating that the tumor had an enhanced optical signal compared with normal tissue background (Fig. 5i). The ex vivo tumor signal intensity was significantly higher than that of the surrounding normal tissue (*P* = 0.0031, Fig. 5j).

The ex vivo optical images of organs or tissue (tumor, heart, liver, spleen, lung, kidney, intestine, brain, muscle, and blood) of mice assigned to TEFI group were acquired (Fig. 5j). The optical signal intensity of the tumor was way much higher than those of other organs, followed by the heart with a faint optical signal. The optical signal of other organs or tissue was extremely weak, indicating an outstanding capability of tumor delineation using this novel technique.

## Discussion

Radical resection is usually difficult to achieve in surgery of malignant tumors, such as breast cancer, glioblastomas, and lung cancer. Therefore, technologies that can assist intraoperative tumor identification are in urgent need. Optical imaging help to identify tumors in real-time during surgery, which leads to high potential for clinical translation. In this study, Gd_2_O_3_:Eu NPs have been combined with a commonly used clinical radiopharmaceutical ^18^F-FDG for outstanding imaging performance. For biomedical use, Gd_2_O_3_:Eu was modified with PVA, and the performance of tumor imaging and image-guided surgery was investigated in small animal models. It is demonstrated on animal models that the mixed ^18^F-FDG and Gd_2_O_3_:Eu-100@PVA performs much better than CLI in tumor imaging and it can be successfully used in image-guided surgery.

When mixed with ^18^F-FDG in vitro, the novel Gd_2_O_3_:Eu-50 NPs achieve a signal intensity 16.19 times higher than that of Eu_2_O_3_, and approximately 369 times higher than that of CL generated by ^18^F-FDG alone. The enhanced signal intensity caused by Gd_2_O_3_:Eu NPs enables in vivo tumor imaging with high contrast. It is also demonstrated that the optical signal intensity decreases as the diameter increases for NPs without PVA modification. This may be affected by the amount of the molecule involved as reported before [32].

The Gd_2_O_3_:Eu NPs have been modified with PVA for improving biocompatibility, which is widely used [33, 34]. With modification, the optical intensity is weakened compared with Gd_2_O_3_:Eu without modification. However, to still take the advantage of high biocompatibility, modified Gd_2_O_3_:Eu was evaluated in the in vivo experiment of tumor imaging and image-guided surgery. Gd_2_O_3_:Eu-100@PVA with a diameter of 100 nm generates an optical signal with the highest intensity among the three modified Gd_2_O_3_:Eu NPs with different diameters. In the experiment of measuring the optical spectrum, the emission light of 700 nm generated by Gd_2_O_3_:Eu-100@PVA is stronger than that of Gd_2_O_3_:Eu-50@PVA and Gd_2_O_3_:Eu-200@PVA. This may be caused by the interaction between PVA and the radiopharmaceutical or the emitted light.

The ex vivo experiments have been performed with Gd_2_O_3_:Eu first. It is demonstrated that the optical signal generated from interaction with CL, β particles, and γ radiation is 33, 40, and 27%, respectively. The experiment using mirror reflection also achieves a similar percentage of interaction with CL. While in the previous study on REFI with Eu_2_O_3_, only 4.6% of the optical signal is generated by CL and 95.4% by γ radiation [26]. This study indicates that Gd_2_O_3_:Eu based nanoparticles are better CL absorbers than Eu_2_O_3_.

The optical spectrum acquired by optical signal using the IVIS spectrum imaging system aligns with that measured by the spectrometer, with a peak of 620 nm and a small peak of 700 nm. Compared with CL with a blue-ultraviolet spectrum, the red-shifted and enhanced light provide deeper penetration and shortened exposure time, which improves the clinical translation potential of TEFI. With the porcine intestine covered on the top of the 96-well plate containing ^18^F-FDG and the NPs, the optical signal of the mixture of ^18^F-FDG and Gd_2_O_3_:Eu is approximately 28 times higher than that of the ^18^F-FDG and Eu_2_O_3_ mixture. This demonstrates the improved tissue penetration capacity with Gd_2_O_3_:Eu combined with ^18^F-FDG. This may enable a reduced dose of NPs used with an outstanding imaging performance, which reduce potential toxicity.

In clinic, PET provides pre-operative imaging on functional and metabolism information of diseases. ^18^F-FDG used in this study is one of the commonly used radiopharmaceuticals for PET in clinic. In this study, the in vivo PET shows no obvious signal of the tumor. While optical images using Gd_2_O_3_:Eu-100@PVA and ^18^F-FDG reveals an obvious optical signal of the tumor. This indicates that the optical imaging of Gd_2_O_3_:Eu-100@PVA and ^18^F-FDG has high potential for tumor detection.

CLI is an emerging optical imaging method that can use radiopharmaceutical for optical imaging. CLI has been applied in intraoperative imaging in 2017 where ex vivo breast tumor tissue samples are obtained intraoperatively and imaged with a specially designed imaging device for CLI, which is used to display the tumor boundary [9]. However, the optical signal intensity of CLI is relatively weak, restricting the real-time intraoperative tumor imaging. Therefore, various methods have been put forward to achieve the enhancement of optical signal intensity. In 2015, Eu_2_O_3_ NP was combined with radiopharmaceuticals in optical imaging, demonstrating a better signal-to-background ratio compared with FMI [26]. As the Eu_2_O_3_ NPs mainly interact with γ radiation, the enhancement of optical signal intensity obtained by the Eu_2_O_3_ NP is still needed to be improved. Besides, the NP without surface modification raises the concern of in vivo toxicity. Another imaging technique, Cerenkov radiation energy transfer (CRET), has been explored using clinical radiopharmaceuticals ^18^F-FDG and ^18^C-choline (^11^C-CHO) together with FDA-approved fluorophore fluorescein sodium (FS). The application of FS further improves the potential of clinical translation as it has been approved for clinical use by the FDA and extensively investigated in different imaging fields. However, FS only translates energy of CL into long-wavelength fluorescence, which does not take full advantages of radiation emitted by radiopharmaceuticals except for CL, such as β particles and γ radiation [18]. In this research, the novel Eu^3+^ doped gadolinium oxide with PVA modification can translate the energy of CL, β particles, and γ radiation generated along with the decay process of ^18^F-FDG. This enhances the optical signal production capability of Gd_2_O_3_:Eu@PVA NP.

The gadolinium-based nanoparticle was reported to have enhanced MRI T1 signal [35, 36]. Therefore, the nanoparticles reported in this article has the potential to be applied as multi-modality tumor imaging. Besides, as fluorescence imaging with light of longer wavelength has shown outstanding performance [37, 38], novel NPs with emission of longer wavelength are of great potential to combine with radiopharmaceuticals for better biomedical use. 

## Conclusion

A novel Eu^3+^ doped gadolinium oxide (Gd_2_O_3_:Eu) is combined with ^18^F-FDG to achieve a red-shifted emitting spectrum and enhanced optical signal intensity. The high conversion efficiency of the radiation energy is realized using the novel NP. Moreover, with PVA modification, Gd_2_O_3_:Eu@PVA with high biocompatibility shows capability for tumor imaging and image-guided surgery in small animal models. Our study highlights that combining Gd_2_O_3_:Eu with ^18^F-FDG greatly integrate the merit of optical imaging and nuclear imaging, worthy of further investigation of more NPs with improved optical properties and biocompatibility for pre-clinical and clinical use.

## Supplementary Information

**Additional file 1**: **Figure 1**. The optical images with different interaction distances and radioactivity.

## Data Availability

The data generated or analyzed during this study are included in the manuscript and the supplementary information files.
