# Supplementary material for: Radiopharmaceutical and Eu3+ doped gadolinium oxide nanoparticles mediated triple-excited fluorescence imaging and image-guided surgery
Source: J Nanobiotechnology. 2021 Jul 16;19:212. doi: 10.1186/s12951-021-00920-6 (PMC8283963; doi:10.1186/s12951-021-00920-6)
Supplement: Supplementary file 1 — Additional file 1: Figure 1. The optical images with different interaction distances and radioactivity. [file 12951_2021_920_MOESM1_ESM.docx]

**Radiopharmaceutical and Eu^3+^ Doped Gadolinium Oxide Nanoparticles mediated Triple-Excited Fluorescence Imaging and Image-Guided Surgery**

Xiaojing Shi^1,2^, Caiguang Cao^1,2^, Zeyu Zhang^1,3^, Jie Tian^1,2,3,*^ , Zhenhua Hu^1,2,*^.

1. CAS Key Laboratory of Molecular Imaging, Beijing Key Laboratory of Molecular Imaging, The State Key Laboratory of Management and Control for Complex Systems, Institute of Automation, Chinese Academy of Sciences, Beijing, China.

2. School of Artificial Intelligence, University of Chinese Academy of Sciences, Beijing, China.

3. Beijing Advanced Innovation Center for Big Data-Based Precision Medicine, School of Medicine, Beihang University, Beijing, China.

**Footnote：**

* Corresponding authors: E-mail: jie.tian@ia.ac.cn (J.T.) and zhenhua.hu@ia.ac.cn (Z.H.)


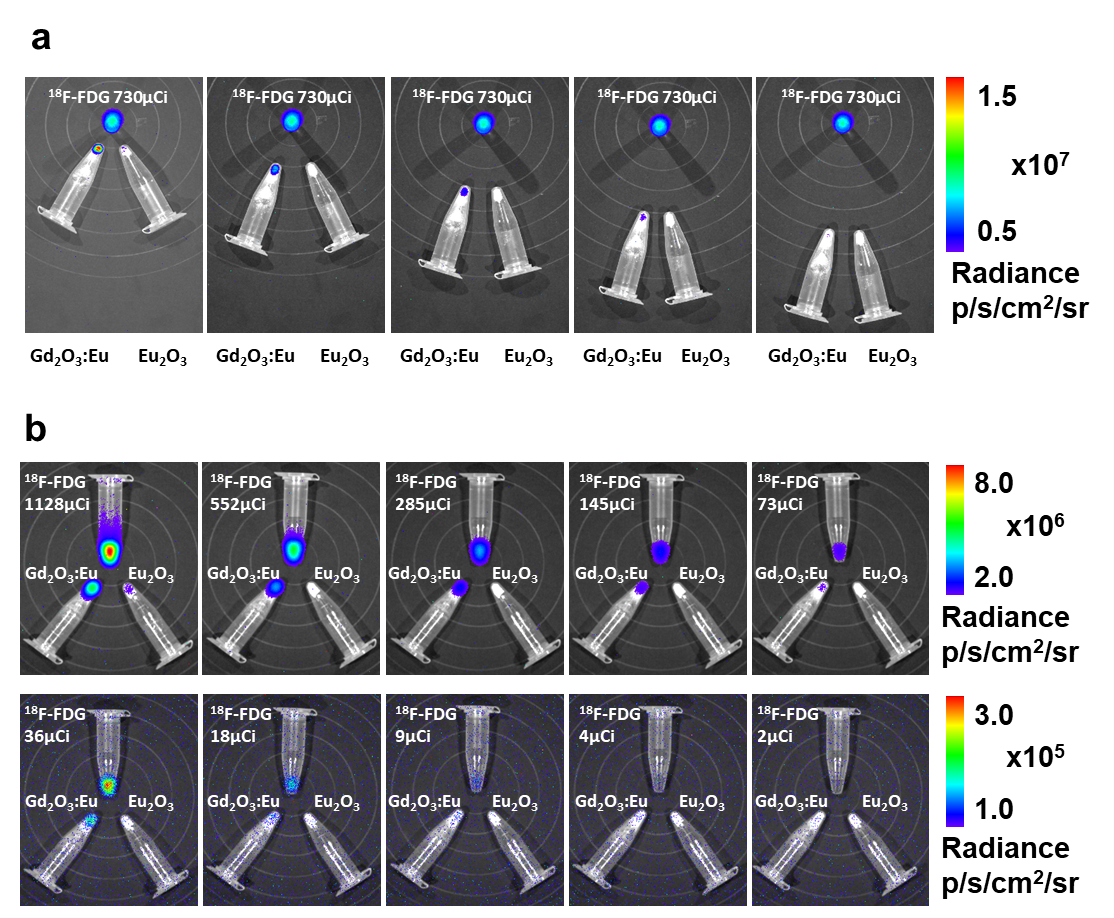


**Supplementary Figure 1.** **the optical images with different interaction distances and radioactivity**. **a,** the impact of interaction distance on optical signal intensity. **b,** the impact of radioactivity on optical signal intensity.
